# Temperature measurements of pacemaker leads in a 1.0T high field open MRI using various MR sequences: initial results

**DOI:** 10.1186/1532-429X-13-S1-P65

**Published:** 2011-02-02

**Authors:** Sebastian A Seitz, Julian J Ebner, Gerriet Petry, Evangelos Giannitsis, Olaf Dössel, Hugo A Katus, Henning Steen

**Affiliations:** 1 Karlsruhe Institute of Technology (KIT), Karlsruhe, Germany; 2 University Heidelberg, Heidelberg, Germany; 3 Heidelberg Health System, Heidelberg, Germany

## Introduction

Magnetic resonance imaging (MRI) is a valuable diagnostic method for many cardiovascular diseases. To date, patients with pacemakers are contra-indicated for cardiac MRI exams due to several effects that can occur during the MRI procedure: a) heating of the lead-tip, and b) less hazardous sensing errors and device malfunctions. Almost all measurements on MRI pacemaker compatibility have been conducted on classic 1.5 or 3T cylindrical whole-body MRI systems. In contrast, this study focused on the use of a high field open MRI (HFO) system due to its advantageous properties of RF fields which are commonly made responsible for the induction of lead heating.

## Purpose

Determine the feasibility of MRI examinations of patients with cardiac pacemakers using an open 1.0 T MRT system and realistic cardiac imaging sequences.

## Methods

**Figure 1:**
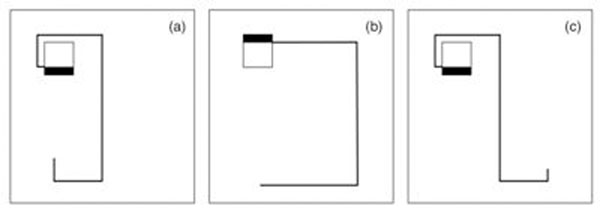
Pacemaker/lead configurations imitating common implantation patterns.

Two high energy (1.+2.) and two realistic clinical cardiac (3.+4.) MR-sequences with artificial ECG-triggering at 60/min were used on the 1.0T HFO system: 1. T2-TSE (TR/TE=177/38ms;TSE-factor=16; time=58s;flip-angle=90°); 2. 3-D bTFE (TR/TE=4.7/2.4ms;TFE-factor=6;time=382s;flip-angle=70°;TFE-shot-duration=34ms;TFE-shots=532), 3. SSFP-cine (TR/TE=4.7/2.2ms;TFE-factor=16; time=90s;flip-angle=70°;TFE-shot-duration=76ms;TFE-shots=76), 4. 3D-FFE multi-shot (inversion-recovery; TR/TE=3.9/1.3ms;TFE-factor=21;time=23s;flip-angle=15°; TFE-shot-duration=157ms;TFE shots=46). Two types of bipolar cardiac pacing leads were evaluated: a) conventional lead (Medtronic Capsurefix Novus), 2. MR compatible lead (Medtronic Capsurefix MRI SureScan) both connected to a St. Jude Frontier II pacemaker. The pacemaker/lead system was placed in a saline-filled Plexiglas phantom imitating three clinically common lead positions (figure1). Temperature measurements were captured with a fiber-optic measurement system.

## Results

**Figure 2:**
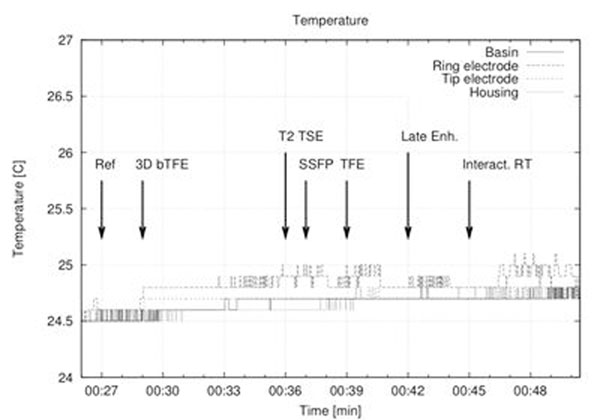
Development of temperature at four different positions (Tip electrode, ring electrode, pacemaker housing, basin/phantom) while exposed to a series for MRI sequences, pacemaker/lead configuration as shown in Fig. 1(a)

The highest temperature increase (0.6 °C) was observed in the (a) configuration when exposed to the realtime interactive sequence (see fig. 2). In none of the utilized scan protocols severe heating could be measured.

## Conclusions

In-vitro measurement of an MR compatible and a regular pacemaker lead in geometrically realistic positions in an HFO open MRI system showed no relevant tip heating for both ECG-gated high energy as well as clinically used cardiac MR sequences. Further in-vivo research has to be conducted to clarify the relevance of these findings.

